# Macular choroidal thickness and peripapillary retinal nerve fiber layer thickness in normal adults and patients with optic atrophy due to acute idiopathic demyelinating optic neuritis

**DOI:** 10.1371/journal.pone.0198340

**Published:** 2018-06-01

**Authors:** Kyung-Ah Park, Daye Diana Choi, Sei Yeul Oh

**Affiliations:** Department of Ophthalmology, Samsung Medical Center, Sungkyunkwan University School of Medicine, Seoul, Korea; Weill Cornell Medicine-Qatar, QATAR

## Abstract

**Purpose:**

To evaluate the association between macular choroidal thickness and peripapillary RNFL thickness in patients with optic atrophy due to acute idiopathic demyelinating optic neuritis and in normal controls using spectral domain optical coherence tomography (SD-OCT).

**Methods:**

We performed SD-OCT peripapillary RNFL circular scan centered on the optic disc with horizontal and vertical crosshair scans through the fovea using the enhanced depth technique in 62 eyes with optic atrophy due to acute idiopathic demyelinating optic neuritis and 86 eyes of normal controls. The association between RNFL thickness and macular choroidal thickness measurements was assessed.

**Results:**

The mean age was 43 ± 14 years (mean ± SD) in patients with optic atrophy and 45 ± 16 years in healthy controls (p = 0.791). There was a significant association between nasal peripapillary RNFL thickness and choroidal thickness at 3.0 mm nasal to the foveal center in patients with optic atrophy in multivariate analysis (estimate = 1.398, p = 0.011). In controls, there were significant associations between global average, superior, and inferior peripapillary RNFL thickness and choroidal thickness at 3.0 mm superior to the foveal center (estimate = -60.112, p = 0.044, estimate = 15.821, p = 7.312, and estimate = 15.203, p = 7.222, respectively).

**Conclusions:**

Our SD-OCT data revealed that there was a significant association between peripapillary RNFL thickness and macular choroidal thickness in patients with optic atrophy due to acute idiopathic demyelinating optic neuritis and in controls, although the mechanism remained unclear. The difference in the pattern of association between patients with optic atrophy and controls suggests that optic atrophy caused by acute idiopathic demyelinating optic neuritis could affect the pattern of association between peripapillary RNFL thickness and macular choroidal thickness.

## Introduction

Optical coherence tomography (OCT) is a noninvasive and objective method for quantitating axonal and neuronal loss in patients with various types of optic neuropathies and disorders of the central nervous system[1–3]. Peripapillary retinal nerve fiber layer analysis has become one of the most useful tools for evaluating optic nerve damage in optic neuropathies. Recently, in addition to measurement of nerve fiber layer thickness, choroidal thickness has been investigated in various optic neuropathies including glaucoma, anterior ischemic optic neuropathy, and optic neuritis (ON)[4–12]. Several studies have reported that peripapillary choroidal thickness is thinner in glaucomatous eyes compared to that in normal eyes[6, 7]. However, the result is controversial[4, 5]. Peripapillary and macular choroidal thicknesses are also related to anterior ischemic optic neuropathy[8–11]. One study has reported that peripapillary RNFL thinning in non-glaucomatous optic atrophy is associated with peripapillary choroidal thinning[12]. Another population-based study on 478 non-glaucomatous adults has revealed that thinner peripapillary choroidal thickness is associated with thinner peripapillary RNFL thickness[11]. However, the association between macular choroidal thickness and peripapillary RNFL thickness in patients with optic atrophy due to ON or in normal controls is currently unclear. Therefore, the objective of this study was to evaluate the association between macular choroidal thickness and peripapillary RNFL thickness in patients with optic atrophy due to acute idiopathic demyelinating ON and in normal controls. Such evaluation might provide a better understanding of the relationship between structural parameters that might be useful for assessing optic nerve damage using OCT in various optic neuropathies.

## Methods

This retrospective study was approved by the Institutional Review Board of Samsung Medical Center (Seoul, Republic of Korea). It was performed at a single center according to the tenets of the Declaration of Helsinki. This study included the following two groups of participants: 46 patients (62 eyes) with optic atrophy caused by acute idiopathic demyelinating ON and 47 healthy controls (86 eyes), who visited the general or neuro-ophthalmology clinic between April 1, 2011 and March 31, 2016. Most of healthy controls were hospital workers who underwent routine ocular examination.

Inclusion criterion for the optic atrophy group was patient who had a clinical episode of acute idiopathic demyelinating ON. A history of acute idiopathic demyelinating ON episodes was confirmed by medical chart review. The diagnosis of an acute idiopathic demyelinating ON episode was based on documented findings of decreased visual acuity, visual field defect, color vision loss, relative afferent pupil defect, pain on eye movements, and a normal fundus or optic disc swelling without other retinal pathology. Patients with any of the following conditions were excluded from this study: age of less than 20 years or greater than 80 years, ON episode within 6 months, refractive error less than -6 diopters or more than +3 diopters (spherical equivalent), and any other ocular pathology that might affect OCT measurements including glaucoma and retinal disease. Any patients with neurologic or systemic disorder that could affect OCT measurement such as multiple sclerosis, neuromyelitis optica, and rheumatologic disorders diagnosed at neurology department before the inclusion of the study were also excluded.

None of the 47 disease-free controls had a history of ocular or neurologic disease. However, a refractive error from -6 diopters to +3.0 diopters (spherical equivalent) was allowed.

All patients and healthy controls underwent full ophthalmologic assessment including visual acuity test, slit lamp biomicroscopy and fundus examination. Before vision testing, all subjects underwent detailed refraction check. Corrected visual acuities were transformed to a logarithmic scale (log MAR) for statistical analysis.

All OCT scans were performed with a Spectralis OCT (Heidelberg Engineering, Vista, CA, USA) that provided 40,000 A-scans per second with 7 μm optical and 3.5 μm digital axial resolution. For each patient, horizontal and vertical OCT scans consisting of 512 A-scans per line were obtained from the fovea. An internal fixation target was used, and the patient’s other eye was covered during scanning. All OCT images were converted to grey scale for better visualization and analysis. We obtained OCT peripapillary RNFL circular scans centered on the optic disc of each patient. We also obtained horizontal and vertical OCT crosshair scans. For each patient, enhanced depth imaging OCT was conducted according to a previously described method[13, 14]. The choroid was imaged by positioning an OCT camera close enough to the eye to obtain an inverted image. All measurements in the current study were performed using a 1:1 micron image. Choroidal thickness was measured using Heidelberg Eye Explorer software (version 1.7.0.0). Subfoveal choroidal thickness was defined as the distance from the hyperreflective line of the subfoveal Bruch’s membrane to the innermost hyperreflective line of the subfoveal chorio-scleral interface. Each measurement was performed at the fovea, 1.0 mm and 3.0 mm nasal, temporal, superior, and inferior to the fovea. Two observers, who were blinded to information about whether a patient had optic atrophy performed these measurements twice for all data, and the mean value of the two measurements was used for analysis.

Wilcoxon rank-sum test was used to compare patients’ age, LogMAR visual acuity, spherical equivalent refractive error, temporal and inferior peripapillary RNFL thickness, and choroidal thickness 3.0 mm nasal to the foveal center. Pearson’s Chi-squared test was used to compare gender between the two groups. The peripapillary RNFL thickness at areas other than temporal and inferior sectors and macular choroidal thickness profiles at areas other than 3.0 mm nasal to the foveal center were compared between patients with optic atrophy and healthy controls using Wilcoxon rank-sum test. Association between macular choroidal thickness profiles and other parameters including peripapillary RNFL thickness was assessed in univariate and multivariate analyses using linear regression model. Age, gender, spherical equivalent refractive error, and variables with p-value of less than 0.2 in univariate analysis were entered into multivariate analysis. P-values were corrected by Bonferroni’s correction due to multiple testing. Interobserver variability and intraobserver repeatability were analyzed using intraclass correlation coefficient (ICC): excellent ICC ≥ 0.75; good, ICC ≥ 0.4; and poor, ICC <0.4. Statistical analyses were performed using SAS version 9.4 (SAS Institute, Cary, NC, USA).

## Results

The mean age was 43 ± 14 years (mean ± SD) in patients with optic atrophy and 45 ± 16 years in healthy controls (p = 0.791). Twenty-nine (63%) patients with optic atrophy and 26 (55%) healthy controls were women. The mean spherical equivalent refractive error was -1.2 ± 2.1 diopters in patients with optic atrophy and -0.6 ± 1.5 diopters in controls (p = 0.144). Peripapillary RNFL thickness measurements and descriptive statistics of functional and vision test are summarized in Table 1. Significant differences were observed between eyes with optic atrophy and control eyes in all clinical parameters and in peripapillary RNFL thicknesses.

**Table 1:** Descriptive statistics (mean ± standard deviation) and statistical comparisons of functional and vision test and peripapillary retinal nerve fiber layer thickness in patients with optic atrophy due to acute idiopathic demyelinating optic neuritis and normal controls.

Variable	Eyes with OA (n = 62)	Control eyes (n = 86)	p-value*
	Mean±SD	Mean±SD	
LogMAR visual acuity	0.4±0.7	0.00±0.00	<0.001
Color vision (Ishihara test score/number of test plate)	0.6±0.4		
Visual field (Mean deviation (dB))†	-9.1±9.6		
Peripapillary RNFL thickness (μm)			
Total	67.9±23.5	98.8±11.4	<0.001‡
Temporal	51.7±21.7	80.1±15.5	<0.001‡
Nasal	46.5±22.6	66.3±13.7	<0.001‡
Superior	86.8±31.9	120.2±20.2	<0.001‡
Inferior	86.6±31.6	128.7±18.9	<0.001‡

OA = optic atrophy; SD = standard deviation

*Wilcoxon rank-sum test

^†^Humphrey Field Analyzer using the 30–2 SITA-standard protocol

^‡^P-values were corrected by Bonferroni’s correction due to multiple testing.

Table 2 presents choroidal thickness values measured at nine locations. There was no significant difference in macular choroidal thickness in all sectors. The ICC of the intraobserver repeatability was more than 0.4 when choroidal thickness was measured at the foveal center, 1.0 mm from the foveal center, and 3.0 mm from the foveal center.

**Table 2:** Comparisons of macular choroidal thicknesses in patients with optic atrophy due to acute idiopathic demyelinating optic neuritis and in normal controls.

Variable		Eyes with OA (n = 62)	Control eyes (n = 86)	p-value*
		Mean±SD	Mean±SD	
**Thickness(μm)**				
**Foveal center**		278.7±94.5	271.7±67.6	0.832
**Inner locations†**				
	Inner temporal	271.7±91.1	268.4±69.8	0.983
	Inner nasal	234.9±84.5	240.4±69.0	0.889
	Inner superior	266.1±92.7	276.8±64.9	0.483
	Inner inferior	266.9±107.0	252.4±78.0	0.549
**Outer locations**^‡^				
	Outer temporal	255.9±100.1	239.5±85.4	0.192
	Outer nasal	135.8±64.3	132.2±59.7	0.776
	Outer superior	276.0±84.7	268.3±79.1	0.614
	Outer inferior	238.4±95.6	233.4±74.5	0.955

OA = optic atrophy; SD = standard deviation

*Two-sample t-test

P-values were corrected by Bonferroni’s correction due to multiple testing.

^†^990 to 1000 μm away from the foveal center.

^‡^2990 to 3000 μm away from the foveal center.

With respect to the association between peripapillary RNFL thickness and macular choroidal thickness, there was a significant association between nasal peripapillary RNFL thickness and choroidal thickness at 3.0 mm nasal to the foveal center (estimate = 0.401, p = 0.024) in univariate analysis among patients with optic atrophy. Associations between other parameters and macular choroidal thickness are shown in S1 Table. There was a significant association between inferior peripapillary RNFL thickness and choroidal thickness at the foveal center (estimate = 1.163, p = 0.038) and at 1.0 mm temporal to the foveal center (estimate = 1.481, p = 0.007) in controls. There was also a significant association between superior peripapillary RNFL thickness and choroidal thickness at 1.0 mm temporal to the foveal center (estimate = 1.189, p = 0.044) and at 3.0 mm inferior to the foveal center (estimate = 1.307, p = 0.032). Associations between other parameters and macular choroidal thickness are shown in S1 Table. Other parameters did not show significant association with macular choroidal thickness.

In multivariate analysis, the association between nasal peripapillary RNFL thickness and choroidal thickness at 3.0 mm nasal to the foveal center remained statistically significant (estimate = 1.398, p = 0.011) in patients with optic atrophy. In addition, spherical equivalent refractive error was significantly associated with choroidal thickness at the foveal center (estimate = 12.881, p = 0.048) in multivariate analysis. Age was significantly associated with choroidal thickness at 1.0 mm superior to the foveal center (estimate = -2.358, p = 0.035) and at 3.0 mm superior to the foveal center (estimate = -2.663, p = 1.108) (S2 Table). In controls, there were significant associations between global average, superior, and inferior peripapillary RNFL thickness and choroidal thickness at 3.0 mm superior to the foveal center (estimate = -60.112, p = 0.044, estimate = 15.821, p = 7.312, and estimate = 15.203, p = 7.222, respectively) in multivariate analysis. In addition, age was significantly associated with choroidal thickness in all sectors in controls (S2 Table). Other parameters did not show significant association with macular choroidal thickness.

## Discussion

In this study, significant differences in peripapillary RNFL thicknesses were observed between eyes with optic atrophy and control eyes, but not in macular choroidal thickness. One previous study has reported that peripapillary choroidal thickness in patients with non-glaucomatous optic atrophy caused by ON is thinner than that in controls globally and at temporal, temporal-superior, and nasal-inferior regions[12]. In our study, there was no significant difference in macular choroidal thickness between patients with optic atrophy and healthy controls. Our data suggest that optic atrophy caused by acute idiopathic demyelinating ON does not induce significant macular choroidal damage which can cause changes in choroidal thickness.

One study has reported that peripapillary RNFL thinning in non-glaucomatous optic atrophy is associated with peripapillary choroidal thinnin[12]. However, no study has reported the association between macular choroidal thickness and peripapillary RNFL thickness in patients with non-glaucomatous optic atrophy or in normal adults up to date. In multivariate analysis of this study, there was a significant association between nasal peripapillary RNFL thickness and choroidal thickness at 3.0 mm nasal to the foveal center in patients with optic atrophy. In controls, there were significant associations between global average, superior, and inferior peripapillary RNFL thickness and choroidal thickness at 3.0 mm superior to the foveal center. Previously, Gupta et al. have suggested that there could be a positive association between peripapillary choroidal thickness and peripapillary RNFL thickness because both the optic nerve head and the peripapillary choroid share a common source of blood supply via short posterior ciliary arteries[11]. If neurodegeneration occurs at the optic nerve head, concurrent choroidal changes could also occur in areas of RNFL thinning. However, it is currently unclear whether the positive association between macular choroidal thickness and peripapillary RNFL thickness in normal controls is due to the common vascular supply between the peripapillary retinal nerve fiber and choroid. One cross-sectional study conducted on 340 healthy children has reported that RNFL thickness was one of the independent factors of macular choroidal thickness[15]. Confounding factors such as age, refractive error, and axial length might also affect both peripapillary RNFL thickness and choroidal thickness. Spherical equivalent refractive error was significantly associated with choroidal thickness at the foveal center only in patients with optic atrophy. Age was significantly associated with choroidal thickness at 1.0 mm superior to the foveal center and at 3.0 mm superior to the foveal center in patients with optic atrophy. In controls, age was significantly associated with choroidal thickness in all sectors. In this study, we did not routinely check axial length. Instead, spherical equivalent refractive error along with age was adjusted in multivariate analysis. However, strong confounding factors might have affected statistical analysis results even after adjustment. Further confirmative study should match age and refractive error or axial length carefully during inclusion of study subjects and controls.

The discrepancy between patients with optic atrophy and controls also suggests that optic atrophy caused by acute idiopathic demyelinating ON could affect the association between peripapillary RNFL thickness and macular choroidal thickness. Although ON is an inflammatory disorder involving neuronal tissues, Wang et al. have previously reported a significantly lower optic nerve flow index in eyes with ON[16]. Those vascular changes could affect the pattern of association between choroidal thicknesses and peripapillary RNFL thicknesses. However, it is currently unclear whether choroidal involvement is a primary event or secondary to RNFL loss. Optic neuropathies do not always induce diffuse optic nerve damage. The pattern of optic nerve damage can be different among different types of optic neuropathies. Previous OCT studies have revealed that the temporal peripapillary quadrant is more greatly affected in ON[17]. Therefore, predilection of optic atrophy, in specific areas of the optic nerve, in ON could affect the association between choroidal thickness and peripapillary RNFL thickness.

This study has several limitations. First, the use of only a single institution might have limited the power of this study’s conclusions. In addition, this study was performed using data from the same ethnic group. Some results may not be valid in other ethnic groups. Thirdly, this study only analyzed optic atrophy caused by ON. Therefore, results may not be directly applied to other types of optic neuropathy. Fourthly, we used arbitrary location for choroidal measurements. Measurements at different location could lead to different results. Lastly, although we excluded patients with neurologic or systemic disorder such as multiple sclerosis, neuromyelitis optica, or rheumatologic disorders diagnosed at neurology department before inclusion of this study, there might be other disorders related to ON that were not diagnosed. For example, we only performed serologic test for neuromyelitis optica without performing study on myelin oligodendrocyte glycoprotein. Therefore, our study participants could have heterogeneous mechanisms of ON and consequent optic atrophy that might have affected our results.

In conclusion, this study revealed that there was a significant association between peripapillary RNFL thickness and macular choroidal thickness in patients with non-glaucomatous optic atrophy and in controls although the mechanism remained unclear. The pattern of association between peripapillary RNFL thickness and macular choroidal thickness was affected by optic atrophy. Further studies including larger populations and different etiologies of optic neuropathies are needed to clearly reveal the association between peripapillary RNFL thickness and macular choroidal thickness and the clinical significance of these parameters.

## Supporting information

S1 Table Association between other parameters and macular choroidal thickness in univariate analysis. (DOCX)

S2 Table Associations between other parameters and macular choroidal thickness in multivariate analysis. (DOCX)
