# Supplementary material for: Macular choroidal thickness and peripapillary retinal nerve fiber layer thickness in normal adults and patients with optic atrophy due to acute idiopathic demyelinating optic neuritis
Source: PLoS One. 2018 Jun 1;13(6):e0198340. doi: 10.1371/journal.pone.0198340 (PMC5983443; doi:10.1371/journal.pone.0198340)
Supplement: S1 Table — (DOCX) [file pone.0198340.s001.docx]

**Table. Association between other parameters and macular choroidal thickness in univariate analysis**

| **Eyes with OA** | Baseline characteristics | Estimate | p-value* | **Control eyes** | Baseline characteristics | Estimate | p-value* |
| --- | --- | --- | --- | --- | --- | --- | --- |
| **Location of choroidal thickness measurement** | |  |  | **Location of choroidal thickness measurement** | |  |  |
| **Foveal center** | |  |  | **Foveal center** | |  |  |
|  | Age | -1.344 | 0.165 |  | Age | -2.001 | 0.002 |
|  | Gender | 47.320 | 0.095 |  | Gender | -36.163 | 0.070 |
|  | SE refractive error | 12.104 | 0.062 |  | SE refractive error |  |  |
|  | LogMAR visual acuity | 6.222 | 0.773 |  |  |  |  |
|  | Color vision (Ishihara test) | -3.488 | 0.394 |  |  |  |  |
|  | Visual field (Mean deviation)† | 0.253 | 0.863 |  |  |  |  |
|  | Number of ON episodes | 13.508 | 0.402 |  |  |  |  |
|  | Peripapillary RNFL thickness |  |  |  | Peripapillary RNFL thickness |  |  |
|  | Global average | -0.317 | 0.599 |  | Global average | 1.566 | 0.113 |
|  | Temporal | -0.837 | 0.198 |  | Temporal | -0.342 | 0.651 |
|  | Nasal | 0.390 | 0.559 |  | Nasal | 0.438 | 0.567 |
|  | Superior | -0.079 | 0.861 |  | Superior | 0.990 | 0.098 |
|  | Inferior | -0.410 | 0.364 |  | Inferior | 1.163 | 0.038 |
| **Inner locations‡** | |  |  | **Inner locations‡** | |  |  |
| Inner temporal | |  |  | Inner temporal | |  |  |
|  | Age | -2.086 | 0.021 |  | Age | -2.323 | <0.001 |
|  | Gender | 43.643 | 0.105 |  | Gender | -21.711 | 0.280 |
|  | SE refractive error | 9.192 | 0.139 |  | SE refractive error | -4.723 | 0.473 |
|  | LogMAR visual acuity | -1.466 | 0.943 |  |  |  |  |
|  | Color vision (Ishihara test) | -2.303 | 0.542 |  |  |  |  |
|  | Visual field (Mean deviation)† | 0.655 | 0.643 |  |  |  |  |
|  | Number of ON episodes | 18.313 | 0.229 |  |  |  |  |
|  | Peripapillary RNFL thickness |  |  |  | Peripapillary RNFL thickness |  |  |
|  | Global average | 0.020 | 0.972 |  | Global average | 2.296 | 0.018 |
|  | Temporal | -0.121 | 0.844 |  | Temporal | 0.209 | 0.781 |
|  | Nasal | 0.291 | 0.643 |  | Nasal | 0.693 | 0.362 |
|  | Superior | 0.155 | 0.713 |  | Superior | 1.189 | 0.044 |
|  | Inferior | -0.185 | 0.422 |  | Inferior | 1.481 | 0.007 |
| Inner nasal | |  |  | Inner nasal | |  |  |
|  | Age | -0.797 | 0.398 |  | Age | -1.717 | 0.008 |
|  | Gender | 46.539 | 0.089 |  | Gender | -33.346 | 0.098 |
|  | SE refractive error | 9.431 | 0.135 |  | SE refractive error | -4.328 | 0.516 |
|  | LogMAR visual acuity | -3.815 | 0.855 |  |  |  |  |
|  | Color vision (Ishihara test) | -0.505 | 0.899 |  |  |  |  |
|  | Visual field (Mean deviation)† | 0.827 | 0.570 |  |  |  |  |
|  | Number of ON episodes | 9.913 | 0.531 |  |  |  |  |
|  | Peripapillary RNFL thickness |  |  |  | Peripapillary RNFL thickness |  |  |
|  | Global average | 0.126 | 0.830 |  | Global average | 0.823 | 0.413 |
|  | Temporal | -0.615 | 0.330 |  | Temporal | -0.981 | 0.194 |
|  | Nasal | 0.895 | 0.162 |  | Nasal | 0.636 | 0.408 |
|  | Superior | 0.157 | 0.717 |  | Superior | 0.448 | 0.462 |
|  | Inferior | 0.016 | 0.972 |  | Inferior | 0.927 | 0.104 |
| Inner superior | |  |  | Inner superior | |  |  |
|  | Age | -2.074 | 0.023 |  | Age | -1.880 | 0.001 |
|  | Gender | 34.144 | 0.213 |  | Gender | -32.289 | 0.064 |
|  | SE refractive error | 9.311 | 0.138 |  | SE refractive error | -4.749 | 0.411 |
|  | LogMAR visual acuity | -2.239 | 0.558 |  |  |  |  |
|  | Color vision (Ishihara test) | -2.239 | 0.558 |  |  |  |  |
|  | Visual field (Mean deviation)† | 0.360 | 0.803 |  |  |  |  |
|  | Number of ON episodes | 14.050 | 0.371 |  |  |  |  |
|  | Peripapillary RNFL thickness |  |  |  | Peripapillary RNFL thickness |  |  |
|  | Global average | -0.166 | 0.776 |  | Global average | 0.474 | 0.588 |
|  | Temporal | -0.385 | 0.542 |  | Temporal | -0.527 | 0.424 |
|  | Nasal | 0.431 | 0.503 |  | Nasal | -0.308 | 0.645 |
|  | Superior | -0.043 | 0.921 |  | Superior | 0.561 | 0.287 |
|  | Inferior | -0.319 | 0.464 |  | Inferior | 0622 | 0.211 |
| Inner inferior | |  |  | Inner inferior | |  |  |
|  | Age | -2.353 | 0.027 |  | Age | -2.308 | 0.001 |
|  | Gender | 69.283 | 0.027 |  | Gender | -40.654 | 0.075 |
|  | SE refractive error | 10.640 | 0.146 |  | SE refractive error | -5.374 | 0.477 |
|  | LogMAR visual acuity | 16.413 | 0.497 |  |  |  |  |
|  | Color vision (Ishihara test) | -4.766 | 0.294 |  |  |  |  |
|  | Visual field (Mean deviation)† | 0.340 | 0.832 |  |  |  |  |
|  | Number of ON episodes | 17.616 | 0.337 |  |  |  |  |
|  | Peripapillary RNFL thickness |  |  |  | Peripapillary RNFL thickness |  |  |
|  | Global average | -0.051 | 0.939 |  | Global average | 0.874 | 0.443 |
|  | Temporal | -0.637 | 0.379 |  | Temporal | -0.782 | 0.363 |
|  | Nasal | 0.647 | 0.380 |  | Nasal | 0.202 | 0.817 |
|  | Superior | 0.144 | 0.773 |  | Superior | 0.528 | 0.444 |
|  | Inferior | -0.244 | 0.627 |  | Inferior | 1.047 | 0.105 |
| **Outer locations^a^** | |  |  | **Outer locations^a^** | |  |  |
| Outer temporal | |  |  | Outer temporal | |  |  |
|  | Age | -2.675 | 0.003 |  | Age | -2.518 | <0.001 |
|  | Gender | 41.518 | 0.129 |  | Gender | -6.164 | 0.777 |
|  | SE refractive error | -0.179 | 0.978 |  | SE refractive error | -7.784 | 0.270 |
|  | LogMAR visual acuity | -27.316 | 0.186 |  |  |  |  |
|  | Color vision (Ishihara test) | 0.885 | 0.816 |  |  |  |  |
|  | Visual field (Mean deviation)† | 2.465 | 0.122 |  |  |  |  |
|  | Number of ON episodes | 6.409 | 0.686 |  |  |  |  |
|  | Peripapillary RNFL thickness |  |  |  | Peripapillary RNFL thickness |  |  |
|  | Global average | 0.686 | 0.235 |  | Global average | 1.729 | 0.102 |
|  | Temporal | 0.637 | 0.309 |  | Temporal | 0.217 | 0.789 |
|  | Nasal | 0.512 | 0.419 |  | Nasal | 0.344 | 0.675 |
|  | Superior | 0.670 | 0.116 |  | Superior | 1.117 | 0.080 |
|  | Inferior | 0.326 | 0.451 |  | Inferior | 1.007 | 0.096 |
| Outer nasal | |  |  | Outer nasal | |  |  |
|  | Age | -0.843 | 0.252 |  | Age | -2.060 | <0.001 |
|  | Gender | 14.978 | 0.491 |  | Gender | -22.445 | 0.219 |
|  | SE refractive error | 7.610 | 0.124 |  | SE refractive error | -4.885 | 0.414 |
|  | LogMAR visual acuity | -8.554 | 0.601 |  |  |  |  |
|  | Color vision (Ishihara test) | 1.248 | 0.688 |  |  |  |  |
|  | Visual field (Mean deviation)† | 1.390 | 0.216 |  |  |  |  |
|  | Number of ON episodes | -4.906 | 0.693 |  |  |  |  |
|  | Peripapillary RNFL thickness |  |  |  | Peripapillary RNFL thickness |  |  |
|  | Global average | 0.527 | 0.248 |  | Global average | 0.754 | 0.404 |
|  | Temporal | -0.017 | 0.972 |  | Temporal | -0.339 | 0.620 |
|  | Nasal | 1.116 | 0.024 |  | Nasal | -0.034 | 0.961 |
|  | Superior | 0.401 | 0.236 |  | Superior | 0.463 | 0.398 |
|  | Inferior | 0.286 | 0.404 |  | Inferior | 0.817 | 0.111 |
| Outer superior | |  |  | Outer superior | |  |  |
|  | Age | -2.524 | 0.001 |  | Age | -2.812 | <0.001 |
|  | Gender | 23.409 | 0.326 |  | Gender | -36.033 | 0.097 |
|  | SE refractive error | 6.831 | 0.211 |  | SE refractive error | -8.414 | 0.239 |
|  | LogMAR visual acuity | -7.633 | 0.671 |  |  |  |  |
|  | Color vision (Ishihara test) | 0.363 | 0.914 |  |  |  |  |
|  | Visual field (Mean deviation)† | 1.802 | 0.141 |  |  |  |  |
|  | Number of ON episodes | 2.943 | 0.832 |  |  |  |  |
|  | Peripapillary RNFL thickness |  |  |  | Peripapillary RNFL thickness |  |  |
|  | Global average | 0.291 | 0.565 |  | Global average | 1.268 | 0.240 |
|  | Temporal | 0.312 | 0.569 |  | Temporal | 0.160 | 0.842 |
|  | Nasal | 0.836 | 0.130 |  | Nasal | -0.760 | 0.359 |
|  | Superior | 0.105 | 0.780 |  | Superior | 1.200 | 0.063 |
|  | Inferior | 0.028 | 0.942 |  | Inferior | 0.992 | 0.106 |
| Outer inferior | |  |  | Outer inferior | |  |  |
|  | Age | -2.572 | 0.004 |  | Age | -2.675 | <0.001 |
|  | Gender | 60.347 | 0.023 |  | Gender | -29.185 | 0.159 |
|  | SE refractive error | 4.430 | 0.481 |  | SE refractive error | -3.102 | 0.650 |
|  | LogMAR visual acuity | 0.703 | 0.973 |  |  |  |  |
|  | Color vision (Ishihara test) | -2.053 | 3.852 |  |  |  |  |
|  | Visual field (Mean deviation)† | 0.297 | 0.828 |  |  |  |  |
|  | Number of ON episodes | -1.635 | 0.917 |  |  |  |  |
|  | Peripapillary RNFL thickness |  |  |  | Peripapillary RNFL thickness |  |  |
|  | Global average | 0.260 | 0.644 |  | Global average | 1.642 | 0.107 |
|  | Temporal | -0.136 | 0.824 |  | Temporal | 0.015 | 0.985 |
|  | Nasal | 0.776 | 0.209 |  | Nasal | 0.119 | 0.881 |
|  | Superior | 0.177 | 0.671 |  | Superior | 1.307 | 0.032 |
|  | Inferior | 0.125 | 0.767 |  | Inferior | 0.940 | 0.107 |
|  |  |  |  |  |  |  |  |
|  |  |  |  |  |  |  |  |
|  |  |  |  |  |  |  |  |
| OA=optic atrophy; SE=Spherical equivalent; ON=optic neuritis | | | | | | | |
| *Linear regression analysis  P-values were corrected by Bonferroni’s correction due to multiple testing.  †Humphrey Field Analyzer using the 30-2 SITA-standard protocol | | | | | | | |
| ‡990 to 1000 ㎛ away from the foveal center. | | | |  |  |  |  |
| ^a^2990 to 3000 ㎛ away from the foveal center. | | | |  |  |  |  |
