# Supplementary material for: Macular choroidal thickness and peripapillary retinal nerve fiber layer thickness in normal adults and patients with optic atrophy due to acute idiopathic demyelinating optic neuritis
Source: PLoS One. 2018 Jun 1;13(6):e0198340. doi: 10.1371/journal.pone.0198340 (PMC5983443; doi:10.1371/journal.pone.0198340)
Supplement: S2 Table — (DOCX) [file pone.0198340.s002.docx]

**Table.** Associations between other parameters and macular choroidal thickness in multivariate analysis

| **Eyes with OA** | Baseline characteristics | Estimate | p-value* | **Control eyes** | Baseline characteristics | | Estimate | p-value* |
| --- | --- | --- | --- | --- | --- | --- | --- | --- |
| **Location of choroidal thickness measurement** | |  |  | **Location of choroidal thickness measurement** | | |  |  |
| **Foveal center** | |  |  | **Foveal center** | | |  |  |
|  | Age | -1.344 | 0.165 |  | Age | | -2.512 | 0.001 |
|  | Gender | 47.320 | 0.095 |  | Gender | | -30.212 | 0.102 |
|  | SE refractive error | 12.104 | 0.062 |  | SE refractive error | | 8.942 | 0.214 |
|  | LogMAR visual acuity | 6.222 | 0.773 |  |  | |  |  |
|  | Color vision (Ishihara test) | -3.488 | 0.394 |  |  | |  |  |
|  | Visual field (Mean deviation)† | 0.253 | 0.863 |  |  | |  |  |
|  | Number of ON episodes | 13.508 | 0.402 |  |  | |  |  |
|  | Peripapillary RNFL thickness |  |  |  |  | |  |  |
|  | Global average | -0.317 | 0.599 |  |  | |  |  |
|  | Temporal | -0.837 | 0.198 |  |  | |  |  |
|  | Nasal | 0.390 | 0.559 |  |  | |  |  |
|  | Superior | -0.079 | 0.861 |  |  | |  |  |
|  | Inferior | -0.410 | 0.364 |  |  | |  |  |
| **Inner locations‡** | |  |  | **Inner locations‡** | | |  |  |
| Inner temporal | |  |  | Inner temporal | | |  |  |
|  | Age | -2.086 | 0.021 |  | Age | | -2.887 | <0.001 |
|  | Gender | 43.643 | 0.105 |  | Gender | | -15.092 | 0.394 |
|  | SE refractive error | 9.192 | 0.139 |  | SE refractive error | | 9.986 | 0.154 |
|  | LogMAR visual acuity | -1.466 | 0.943 |  |  | |  |  |
|  | Color vision (Ishihara test) | -2.303 | 0.542 |  |  | |  |  |
|  | Visual field (Mean deviation)† | 0.655 | 0.643 |  |  | |  |  |
|  | Number of ON episodes | 18.313 | 0.229 |  |  | |  |  |
|  | Peripapillary RNFL thickness |  |  |  |  | |  |  |
|  | Global average | 0.020 | 0.972 |  |  | |  |  |
|  | Temporal | -0.121 | 0.844 |  |  | |  |  |
|  | Nasal | 0.291 | 0.643 |  |  | |  |  |
|  | Superior | 0.155 | 0.713 |  |  | |  |  |
|  | Inferior | -0.185 | 0.422 |  |  | |  |  |
| Inner nasal | |  |  | Inner nasal | | |  |  |
|  | Age | -0.797 | 0.398 |  | Age | | -2.366 | 0.002 |
|  | Gender | 46.539 | 0.089 |  | Gender | | -24.069 | 0.206 |
|  | SE refractive error | 9.431 | 0.135 |  | SE refractive error | | 3.615 | 0.621 |
|  | LogMAR visual acuity | -3.815 | 0.855 |  |  | |  |  |
|  | Color vision (Ishihara test) | -0.505 | 0.899 |  |  | |  |  |
|  | Visual field (Mean deviation)† | 0.827 | 0.570 |  |  | |  |  |
|  | Number of ON episodes | 9.913 | 0.531 |  |  | |  |  |
|  | Peripapillary RNFL thickness |  |  |  | Peripapillary RNFL thickness | |  |  |
|  | Global average | 0.126 | 0.830 |  |  | |  |  |
|  | Temporal | -0.615 | 0.330 |  | Temporal | | -1.575 | 0.031 |
|  | Nasal | 0.895 | 0.162 |  |  | |  |  |
|  | Superior | 0.157 | 0.717 |  |  | |  |  |
|  | Inferior | 0.016 | 0.972 |  |  | |  |  |
| Inner superior | |  |  | Inner superior | | |  |  |
|  | Age | -2.074 | 0.023 |  | Age | | -2.158 | 0.001 |
|  | Gender | 34.144 | 0.213 |  | Gender | | -29.376 | 0.066 |
|  | SE refractive error | 9.311 | 0.138 |  | SE refractive error | | 4.782 | 0.438 |
|  | LogMAR visual acuity | -2.239 | 0.558 |  |  | |  |  |
|  | Color vision (Ishihara test) | -2.239 | 0.558 |  |  | |  |  |
|  | Visual field (Mean deviation)† | 0.360 | 0.803 |  |  | |  |  |
|  | Number of ON episodes | 14.050 | 0.371 |  |  | |  |  |
|  | Peripapillary RNFL thickness |  |  |  |  | |  |  |
|  | Global average | -0.166 | 0.776 |  |  | |  |  |
|  | Temporal | -0.385 | 0.542 |  |  | |  |  |
|  | Nasal | 0.431 | 0.503 |  |  | |  |  |
|  | Superior | -0.043 | 0.921 |  |  | |  |  |
|  | Inferior | -0.319 | 0.464 |  |  | |  |  |
| Inner inferior | |  |  | Inner inferior | | |  |  |
|  | Age | -2.353 | 0.027 |  | Age | | -2.683 | 0.002 |
|  | Gender | 69.283 | 0.027 |  | Gender | | -36.635 | 0.086 |
|  | SE refractive error | 10.640 | 0.146 |  | SE refractive error | | 6.468 | 0.433 |
|  | LogMAR visual acuity | 16.413 | 0.497 |  |  | |  |  |
|  | Color vision (Ishihara test) | -4.766 | 0.294 |  |  | |  |  |
|  | Visual field (Mean deviation)† | 0.340 | 0.832 |  |  | |  |  |
|  | Number of ON episodes | 17.616 | 0.337 |  |  | |  |  |
|  | Peripapillary RNFL thickness |  |  |  |  | |  |  |
|  | Global average | -0.051 | 0.939 |  |  | |  |  |
|  | Temporal | -0.637 | 0.379 |  |  | |  |  |
|  | Nasal | 0.647 | 0.380 |  |  | |  |  |
|  | Superior | 0.144 | 0.773 |  |  | |  |  |
|  | Inferior | -0.244 | 0.627 |  |  | |  |  |
| **Outer locations^a^** | |  |  | **Outer locations^a^** | | |  |  |
| Outer temporal | |  |  | Outer temporal | | |  |  |
|  | Age | -2.675 | 0.003 |  | Age | | -3.009 | <0.001 |
|  | Gender | 41.518 | 0.129 |  | Gender | | -0.503 | 0.979 |
|  | SE refractive error | -0.179 | 0.978 |  | SE refractive error | | 8.777 | 0.253 |
|  | LogMAR visual acuity | -27.316 | 0.186 |  |  | |  |  |
|  | Color vision (Ishihara test) | 0.885 | 0.816 |  |  | |  |  |
|  | Visual field (Mean deviation)† | 2.465 | 0.122 |  |  | |  |  |
|  | Number of ON episodes | 6.409 | 0.686 |  |  | |  |  |
|  | Peripapillary RNFL thickness |  |  |  |  | |  |  |
|  | Global average | 0.686 | 0.235 |  |  | |  |  |
|  | Temporal | 0.637 | 0.309 |  |  | |  |  |
|  | Nasal | 0.512 | 0.419 |  |  | |  |  |
|  | Superior | 0.670 | 0.116 |  |  | |  |  |
|  | Inferior | 0.326 | 0.451 |  |  | |  |  |
| Outer nasal | |  |  | Outer nasal | | |  |  |
|  | Age | -0.843 | 0.252 |  | Age | | -2.478 | <0.001 |
|  | Gender | 14.978 | 0.491 |  | Gender | | -17.684 | 0.280 |
|  | SE refractive error | 7.610 | 0.124 |  | SE refractive error | | 7.358 | 0.252 |
|  | LogMAR visual acuity | -8.554 | 0.601 |  |  | |  |  |
|  | Color vision (Ishihara test) | 1.248 | 0.688 |  |  | |  |  |
|  | Visual field (Mean deviation)† | 1.390 | 0.216 |  |  | |  |  |
|  | Number of ON episodes | -4.906 | 0.693 |  |  | |  |  |
|  | Peripapillary RNFL thickness |  |  |  |  | |  |  |
|  | Global average | 0.527 | 0.248 |  |  | |  |  |
|  | Temporal | -0.017 | 0.972 |  |  | |  |  |
|  | Nasal | 1.116 | 0.024 |  |  | |  |  |
|  | Superior | 0.401 | 0.236 |  |  | |  |  |
|  | Inferior | 0.286 | 0.404 |  |  | |  |  |
| Outer superior | |  |  | Outer superior | | |  |  |
|  | Age | -2.524 | 0.001 |  | Age | | -3.193 | <0.001 |
|  | Gender | 23.409 | 0.326 |  | Gender | | -32.074 | 0.081 |
|  | SE refractive error | 6.831 | 0.211 |  | SE refractive error | | 6.613 | 0.353 |
|  | LogMAR visual acuity | -7.633 | 0.671 |  |  | |  |  |
|  | Color vision (Ishihara test) | 0.363 | 0.914 |  |  | |  |  |
|  | Visual field (Mean deviation)† | 1.802 | 0.141 |  |  | |  |  |
|  | Number of ON episodes | 2.943 | 0.832 |  |  | |  |  |
|  | Peripapillary RNFL thickness |  |  |  |  | |  |  |
|  | Global average | 0.291 | 0.565 |  |  | |  |  |
|  | Temporal | 0.312 | 0.569 |  |  | |  |  |
|  | Nasal | 0.836 | 0.130 |  |  | |  |  |
|  | Superior | 0.105 | 0.780 |  |  | |  |  |
|  | Inferior | 0.028 | 0.942 |  |  | |  |  |
| Outer inferior | |  |  | Outer inferior | | |  |  |
|  | Age | -2.572 | 0.004 |  | Age | | -3.503 | <0.001 |
|  | Gender | 60.347 | 0.023 |  | Gender | | -19.214 | 0.251 |
|  | SE refractive error | 4.430 | 0.481 |  | SE refractive error | | 14.673 | 0.029 |
|  | LogMAR visual acuity | 0.703 | 0.973 |  |  | |  |  |
|  | Color vision (Ishihara test) | -2.053 | 3.852 |  |  | |  |  |
|  | Visual field (Mean deviation)† | 0.297 | 0.828 |  |  | |  |  |
|  | Number of ON episodes | -1.635 | 0.917 |  |  | |  |  |
|  | Peripapillary RNFL thickness |  |  |  |  | |  |  |
|  | Global average | 0.260 | 0.644 |  |  | |  |  |
|  | Temporal | -0.136 | 0.824 |  |  | |  |  |
|  | Nasal | 0.776 | 0.209 |  |  | |  |  |
|  | Superior | 0.177 | 0.671 |  |  | |  |  |
|  | Inferior | 0.125 | 0.767 |  |  | |  |  |
|  |  |  |  |  |  | |  |  |
| OA=optic atrophy; SE=Spherical equivalent; ON=optic neuritis | | | | | | | | |
| *Linear regression analysis  P-values were corrected by Bonferroni’s correction due to multiple testing.  †Humphrey Field Analyzer using the 30-2 SITA-standard protocol | | | | | | | | |
| ‡990 to 1000 ㎛ away from the foveal center. | | | |  | |  |  |  |
| ^a^2990 to 3000 ㎛ away from the foveal center. | | | |  | |  |  |  |
|  | | | | | | | | |
